# The Impact of Cognitive and Physical Effort Exertion on Physical Effort Decisions: A Pilot Experiment

**DOI:** 10.3389/fpsyg.2021.645037

**Published:** 2021-11-02

**Authors:** Sven van As, Debby G. J. Beckers, Sabine A. E. Geurts, Michiel A. J. Kompier, Masud Husain, Harm Veling

**Affiliations:** ^1^Behavioural Science Institute, Radboud University, Nijmegen, Netherlands; ^2^Nuffield Department of Clinical Neurosciences, University of Oxford, Oxford, United Kingdom

**Keywords:** cognitive fatigue, motivation, effort-based decision-making, physical activity, exercise psychology

## Abstract

Research suggests that cognitive fatigue has a negative impact on physical activity participation. However, the mechanisms underlying this effect are yet unclear. Using an effort-based decision-making paradigm, we examined whether individuals weigh physical effort-costs more strongly when they are cognitively or physically fatigued. Twenty university students visited the lab on three occasions. On each visit, participants underwent a manipulation that was designed to either induce cognitive fatigue (i.e., 2-back task), physical fatigue (i.e., handgrip exercise), or served as a control condition (i.e., documentary watching). After the manipulations, participants performed an effort-based decision-making task in which they decided for 125 offers whether they accepted the offer to exert the required level of physical effort to obtain rewards that varied in value. The probability to accept offers declined with increasing effort requirements whereas the general probability to accept offers was not reduced by any of the experimental conditions. As expected, the decline in accepted offers with increasing effort requirements was stronger after prolonged exertion of physical effort compared to the control condition. Unexpectedly, this effect was not found after exerting cognitive effort, and exploratory analyses revealed that the impact of physical effort exertion on physical effort-based decisions was stronger than that of cognitive effort exertion. These findings suggest that people weight future physical effort-costs more strongly after exerting physical effort, whereas we could not find any evidence for this after exerting cognitive effort. We discuss multiple explanations for this discrepancy, and outline possibilities for future research.

## Introduction

Participation in sufficient physical activity is paramount for health and well-being (Strain et al., 2020). However, inactivity levels in high-income western countries have increased from 31.6% in 2001 to 36.8% in 2016 (Guthold et al., 2018) and insufficient physical activity remains one of the leading causes of non-communicable diseases worldwide. Interestingly, many individuals not meeting the recommended levels of physical activity would like to be more active (Rhodes and De Bruijn, 2013). It is estimated that only 54% of people who intend to be physically active actually achieve their goal. Advancing our understanding of the psychological barriers for engaging in physical activity therefore is of vital relevance.

A growing body of literature suggests that cognitive effort exertion and cognitive fatigue negatively affect physical activity behavior (Van Cutsem et al., 2017b; Brown et al., 2020). Cognitive fatigue is a complex psychobiological state resulting from cognitive effort exertion and is characterized by feelings of low energy, low positive affective states and a reduced motivation to exert effort (van der Linden, 2011; Hockey, 2013). Importantly, it is expected that cognitive fatigue not only reduces motivation for cognitive effort but also for physical effort (Martin et al., 2018; Müller and Apps, 2019). While previous studies indeed find negative effects of prior cognitive exertion and fatigue on subsequent physical *behavior* (Van Cutsem et al., 2017b; Brown et al., 2020), previous studies did not find evidence for a reduced *motivation* for exerting physical effort when being fatigued after performing cognitively demanding tasks (Van Cutsem et al., 2017b). However, these studies used self-reports to assess motivation, which are inherently limited by participants’ ability and willingness to express this motivation accurately (Chong et al., 2016; Massar et al., 2018; Brown and Bray, 2019). Therefore, the motivational consequences of prior cognitive effort exertion (and consequential fatigue) for subsequent physical behavior require additional examination.

Examining effort-based decision-making provides an alternative approach to uncover potential motivational consequences of cognitive fatigue for physical activity participation. Specifically, Müller and Apps (2019) suggest that fatigue modulates the cost-benefit analyses underlying the decision to exert future effort. The costs of effort are expected to weigh more heavily within the cost-benefit trade-off when someone is fatigued (Kanfer, 2011), which reduces the probability to engage in effortful activities (Müller and Apps, 2019). This motivational consequence of fatigue is thought to cross-domains (Müller and Apps, 2019), meaning that cognitive fatigue changes the decision-making process for both cognitive and physical effort. Thus, fatigue has been characterized by a trans-domain intolerance of effort (i.e., “the intolerance of *any* effort,” Thorndike, 1914), which could explain why cognitive fatigue may negatively affect subsequent decisions to engage in physical behavior (cf. see Marcora, 2010, Pageaux, 2014; Martin et al., 2018 for alternative approaches focusing on changes in the *perception* of effort in a fatigued state).

Recent studies tapping into the effort-based decision-making process for physical behavior in a fatigued state provide preliminary support for changes in cost-benefit analyses. Brown and Bray (2019) showed that after a cognitively fatiguing task, participants intended to perform (and actually performed) a subsequent cycling exercise at lower intensities than after watching a documentary. Cognitive fatigue may have led to an increase in the perception of effort (Marcora, 2010; Pageaux, 2014), a reduced willingness to exert effort (Müller and Apps, 2019), or both (Martin et al., 2018). Furthermore, Harris and Bray (2019, 2021) showed that after a cognitively demanding Stroop task, the self-reported cost-benefit balance for a subsequent cycling task turned out more negatively than after watching a documentary, and this reduced the probability that participants chose to cycle. Finally, Iodice et al. (2017) showed that participants’ preferences for low-effort activities were stronger when they were *physically* fatigued compared to a control condition, which implies that physical fatigue made people more sensitive to the perceived costs of future effort. Together, these studies seem to point at the importance of effort-costs for future physical tasks when investigating the impact of fatigue on subsequent physical behavior.

However, some elements of previous studies prohibit definite conclusions about the role of effort-based decision making and changes in cost-benefit analyses in a fatigued state. To date, the assessment of effort-based decisions has been examined for a single choice for physical activity (Brown and Bray, 2019; Harris and Bray, 2019, 2021), which does not enable researchers to assess the avoidance of specific effort costs when fatigued. Moreover, cost-benefit scores were obtained using self-report scales (i.e., Harris and Bray, 2019, 2021). Most studies thus missed the opportunity to assess cost-benefit trade-offs without being limited by participants’ ability and willingness to express their motivation accurately (Chong et al., 2016). An exception comes from Iodice et al. (2017), who employed an actual effort-based decision paradigm in which physical effort was operationalized as task duration. However, in this case, the researchers exclusively focused on the impact of *physical* fatigue on the decisions for physical effort. Thus, it remains unclear what the consequences of cognitive fatigue are for physical effort-based decision-making.

Therefore, we aimed to investigate the impact of cognitive fatigue and physical fatigue on the subsequent decision-making process for exerting physical effort. Note that although we were primarily interested in the effects of cognitive fatigue on decision-making for exerting physical effort, we also included a condition meant to influence physical fatigue to validate the effort-based decision task, and to compare cognitive with physical fatigue. We examined effort-based decision making with a consequential choice task in which participants needed to indicate whether they accepted offers to exert a certain amount of effort for a certain reward. Similar procedures have been extensively tested in animal and human subjects, and such effort-based decisions are considered a valid way to assess motivation to exert effort (for overviews, see Chong et al., 2016; Pessiglione et al., 2018). Furthermore, such effort-based decision-making tasks are interesting to use in the domain of fatigue (Massar et al., 2018), because they allow for repeated consequential decisions within individuals that are not contaminated by actually performing the effortful behaviors (e.g., by informing participants that they will be asked to execute a selection of their decisions after the decision task has ended; Bonnelle et al., 2015; Iodice et al., 2017; Le Heron et al., 2018).

We expected that increments in physical effort requirements of offers would reduce individuals’ probability to accept offers (i.e., main effect of effort requirement; **hypothesis 1**; Hull, 1943). Moreover, we hypothesized that experimentally manipulated cognitive fatigue would negatively influence individuals’ probability to accept offers, independent of the effort requirements (main effect of cognitive fatigue condition; **hypothesis 2a**; Martin et al., 2018; Müller and Apps, 2019). Similarly, we expected that also physical fatigue would negatively affect participants’ probability to accept physically effortful offers (main effect of physical fatigue condition; **hypothesis 2b**; Müller and Apps, 2019). Most important, we expected an interaction between fatigue condition and effort requirement such that the negative effect of effort requirements on the probability to accept offers would be stronger in the cognitive fatigue condition (**hypothesis 3a**; Martin et al., 2018; Müller and Apps, 2019) and the physical fatigue condition (**hypothesis 3b**; Iodice et al., 2017) compared to the control condition^1^.

## Materials and Methods

### Participants

University students were recruited through the research participation system of Radboud University. Eligibility criteria for participation included to be 18--25 years old, having Dutch, English or German as mother tongue, and having at least moderate understanding of the English language. For practical reasons, we preregistered to test a convenience sample within a specific timeframe (November 4th, 2019 until January 1st, 2020; for preregistration, see https://osf.io/zp7te/). Twenty university students participated in our study within this period, of which 17 provided full data (i.e., three sessions, see Procedure and Materials for details), 2 provided data for the first two sessions and one participant provided data for the first session only. This sample size was identical to that of Iodice et al. (2017), who investigated similar effects. The sample consisted of 17 women and 3 men (*M*_age_ = 20, range: 18–25) and were either German (*n* = 14), Dutch (*n* = 5), or English (*n* = 1). Participants were instructed to refrain from drinking alcohol in the 24 h before testing and from caffeine consumption on testing days. Written consent was obtained from all participants and participation was rewarded with course credits and a performance-dependent lottery in which participants could win a Fitbit.

### Procedure and Materials

**FIGURE 1:**
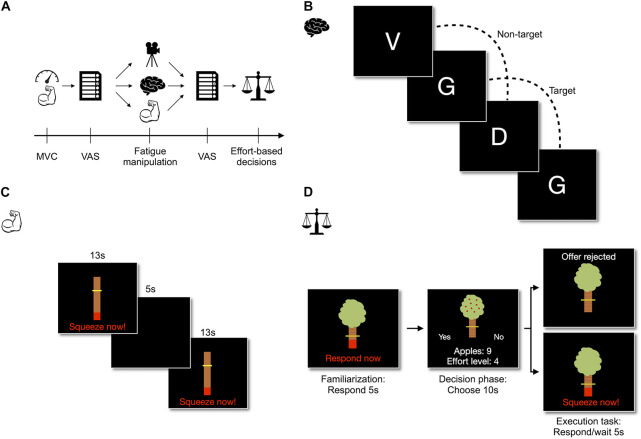
Experimental and task procedures. MVC, Maximum Voluntary Contraction; VAS, Visual Analog Scales measuring cognitive fatigue and physical fatigue. **(A)** Participants visited the lab on three occasions. In each visit, participants underwent one of the three fatigue manipulations (i.e., documentary watching, 2-back task or handgrip exercise) before and after which subjective cognitive and physical fatigue were assessed on a single-item VAS-scale. At the end of each visit, participants performed the effort-based decision-making task. **(B)** In the 2-back task, participants responded to letters appearing on the computer screen. Participants indicated whether the presented letter was the same as two letters before by pressing the corresponding key on a keyboard (“Z” = target, “M” = non-target). **(C)** In the 45-min handgrip exercise, participants squeezed at the required force level for 13 s after which they had a short break of 5 s. **(D)** The effort-based decision-making task consisted of three subtasks. In the familiarization task, participants squeezed at the different force levels (16–80% of MVC) for 5 s. In the decision-making task, participants indicated whether they accepted the offer by pressing the left or right arrow key on the keyboard (position of “yes” and “no” varied per trial). In the execution task, participants performed 40% of the choices made in the decision-making trials. They either squeezed at the required force level for 5 s to obtain the reward of accepted offers or waited for 5 s on the rejected offers. On all squeezing tasks (handgrip exercise, familiarization task and execution task), the yellow bar represented the required force level while the red filling indicated the force participants were currently delivering. Panel D: Adapted from Le Heron et al. (2018). CC BY 4.0.

The experiment had a counterbalanced^2^ within-subject design which consisted of three lab visits on three separate days, with a recovery period of at least 48 h between each visit. Figure 1A provides an overview of the procedure. On each day, participants’ maximum voluntary contraction (MVC) was determined by squeezing in a tailor-made dynamometer three times for 5 s, as hard as they could. Next, participants underwent one of the three experimental conditions for 45 min: cognitive fatigue, physical fatigue or the control condition. Before and after each manipulation, subjective cognitive fatigue and physical fatigue were assessed. Following the manipulations, participants performed a familiarization session in which they experienced different physical effort levels. After several practice trials, they performed an effort-based decision-making task in which they were required, on a trial-by-trial basis, to decide whether they would perform a particular physical effort for a particular magnitude of reward (details below). During this assessment, they did not yet perform the effort because the aim of the effort-based decision-making assessment was to obtain an index of their decisions uncontaminated by physical exertion during the task. To ensure valid decisions, the decision task was made consequential. That is, participants actually performed a representative selection of 40% of their choices after the decision task (i.e., all 25 unique combinations of reward and effort were selected randomly twice) and were informed about this procedure before the decision-making task. After completing all three sessions, participants were debriefed and reimbursed. This procedure has been reviewed and approved by the ethics committee of Radboud University (ECSW-2019-118) and the hypotheses and analyses were preregistered before data collection on the Open Science Framework (for preregistration, see https://osf.io/zp7te/).

#### Fatigue Manipulations

To induce *cognitive fatigue*, participants performed the 2-back working memory paradigm (Kirchner, 1958) for 45 min (see Figure 1B). During the task, individual letters appeared on the computer screen and participants had to indicate whether the current letter was the same as the letter two trials before (i.e., 2-back). Each letter was presented for 500 ms, followed by an inter-trial-interval of 2,500 ms. Before the next letter appeared, participants should respond by pressing either “Z” (2-back) or “M” (no 2-back) on a qwerty-keyboard. The task consisted of 880 trials with target letters being present on 25% of the trials. All letters (“B,” “C,” “D,” “E,” “G,” “J,” “P,” “T,” “V,” and “W”) were presented in capitalized white, Times New Roman against a black background. Before the actual task started, participants received instructions on screen and performed a brief practice session consisting of 32 trials. The 2-back task has been used to induce cognitive fatigue in previous research (e.g., Hopstaken et al., 2015, 2016).

*Physical fatigue* was induced with an intermittent hand-grip exercise (Hilty et al., 2011) of 45 min (see Figure 1C). Similar to the procedures of Iodice et al. (2017), the task that was used to induce physical fatigue thus strongly resembled the physical effort about which participants made effort-based decisions (see below). Participants were instructed to squeeze the dynamometer for 13 s while delivering a required level of grip force as indicated by an interactive computer display and then waited for 5 s until the next trial started. On the first trial, the required grip force was set to 30% of each participants MVC and increased by 10% after two consecutive successes, or decreased by 10% after two consecutive failures. This way, the task was physically fatiguing for all participants while it also ensured that participants could deliver the required force levels throughout the task (i.e., with increasing muscle fatigue). In total, the task consisted of 120 trials, divided over three blocks that were separated by a 45-min break.

In the *control condition*, participants watched the 45-min documentary “Planet Earth—From Pole to Pole” (Fothergill et al., 2006). Documentary watching is frequently used as a control condition in fatigue research (e.g., Marcora et al., 2009; Radstaak et al., 2011; Van Cutsem et al., 2017a) and this specific documentary was chosen as it could be presented in each participant’s mother tongue (i.e., Dutch, German or English). To stimulate engagement, participants were informed that after watching the documentary, they would be asked to indicate for several screenshots whether it was taken from the documentary or not.

Directly before and after each manipulation, participants reported their subjective cognitive and physical fatigue on two single-item VAS-scales (ranging from “Not at all” to “Extremely”: “How mentally/physically fatigued do you currently feel?”). To enhance task motivation, participants were informed they took part in a lottery for winning a Fitbit and that their chances of winning depended on task performance on each of the three manipulation tasks. Specifically, with each successful trial or response, participants increased their chances of winning the Fitbit. Participants did not receive performance feedback during or after the experimental sessions to prevent consequences of (perceived) good or bad performance. This specific procedure was selected to ensure that each trial of each experimental condition was considered equally important for winning the Fitbit.

#### Effort-Based Decision-Making

To quantify participants’ decision-making for physical effort, an adapted version of the accept/reject paradigm (Bonnelle et al., 2015; Le Heron et al., 2018) was used (see Figure 1D). In this task, participants repeatedly chose to accept or reject offers consisting of varying levels of rewards and physical effort. For each offer, participants decided whether they were willing to invest the required level of physical effort to obtain the reward. These offers were visually presented as an apple tree, with a yellow bar on the tree trunk representing the physical effort level (16, 32, 48, 64, or 80% of the participant’s MVC), and the number of apples hanging in the tree representing the rewards (1, 3, 6, 9, or 12 apples). The task consisted of 25 unique offers (i.e., 5 effort levels × 5 reward levels) and each unique offer was presented 5 times, resulting in 125 trials that were divided over 5 blocks. These trials were presented to participants in the exact same random order to prevent between-participant and between-session differences in trial order affecting choice behavior. On each trial, participants had 10 s to indicate whether they accepted the offer by pressing either the left or right arrow-key (key definition varied on a trial-by-trial basis to prevent response biases). Participants also learned that the amount of money they could earn with their decisions depended upon the number of apples they would collect by exerting the trial-based amount of effort during a subsequent execution task in which they would receive a selection of their decisions. Furthermore, they were only informed about the exact value of apples when they received their earnings after completing all test sessions (Bonnelle et al., 2015; Le Heron et al., 2018). This was done to control possible individual differences in weighing of the absolute reward value.

Right before the decision-making task, participants performed a familiarization session in which they actually experienced the five levels of physical effort by squeezing the dynamometer at the required force levels twice. They also completed a practice block of 18 trials to get used to the decision-making procedure. Following the decision-making task, participants performed the execution task, which consisted of 50 trials that were drawn from the decision-making task (40% of trials). Specifically, each unique combination of effort and reward was selected twice, once from the first and once from the second half of the decision-making task. Depending on the choices made during the decision-making task, they could either squeeze the dynamometer for 5 s at the required force level to obtain the apples (i.e., accepted offers), or wait for 5 s until the next trial started (i.e., rejected offers). During this execution phase, participants could not change the choices made earlier during the decision task (e.g., squeezing on a previously rejected trial did not lead to any reward). The execution phase thus only served to increase validity of the decision-making task and these execution data were not analyzed for answering the research question. Each gathered apple represented 1/3 eurocent, which meant that participants could earn up to €3 extra in total.

### Analysis

Data were first screened for invalid trials on which participants gave an erroneous response (i.e., a different key than the response keys) or no response at all. All analyses were performed in the statistical programming software R (R Core Team, 2020). In line with our preregistration, all hypotheses were tested using (generalized) linear mixed-effects models [(G)LMM] with the (g)lmer function (lme4 package; version 1.1-23; Bates et al., 2015). Following the advice of Barr et al. (2013), we used a maximal random effects structure to prevent inflation of Type I errors. Robust *p*-values were obtained with Type III bootstrapped Likelihood Ratio tests using the “mixed” function (afex package; version 0.27-2; Singmann et al., 2015). *Post hoc* tests were performed with the “emmeans” function (emmeans package; version 1.4.4; Lenth, 2020) or by re-testing the GLMM within the conditions of interest. Zero-sum coding was used for all factorial predictors.

#### Manipulation Checks

To investigate whether the manipulations had their intended effects, we ran an LMM testing whether participants experienced the cognitive fatigue condition to be more cognitively fatiguing than the physical fatigue condition and the control condition^3^. The model included a fixed intercept and fixed effects for condition (cognitive fatigue, physical fatigue, control), time (pre, post) and the interaction term Condition × Time. In addition, the model included a per-participant random adjustment to the fixed intercept (i.e., “random intercept”) as well as to the fixed slope of time (i.e., “random slope”). *Post hoc* analyses were performed to investigate which conditions differed from one another.

Second, we investigated whether participants experienced the physical fatigue condition to be more physically fatiguing than the cognitive fatigue condition and the control condition. The model included a fixed intercept and fixed effects for condition (cognitive fatigue, physical fatigue, control), time (pre, post) and the interaction term Condition × Time. In addition, the model included a per-participant random adjustment to the fixed intercept as well as to the fixed slopes of cognitive fatigue and time. *Post hoc* analyses were performed to compare the specific experimental conditions.

Finally, two exploratory analyses (i.e., not preregistered) were performed to obtain insight into participants’ performance on the 2-back task. These analyses and outcomes can be found in Supplementary Material.

#### Main Analyses

To investigate to what extent the probability to accept offers during the decision-making task was influenced by the effort requirements (hypothesis 1), the fatigue manipulations (hypothesis 2a and 2b), or the interaction between the fatigue manipulations and the physical effort requirements (i.e., physical effort slope per condition; hypothesis 3a and 3b), we ran a GLMM^4^. The model included a fixed intercept and fixed slopes for the within-subject factors condition (cognitive fatigue, physical fatigue, control), physical effort requirement (continuous) and the interaction term Condition × Physical Effort Requirement^5^. In addition, a per-participant random adjustment to the fixed intercept as well as per-participant random adjustments to the fixed effects were included in the model. *Post hoc* analyses were performed to investigate which specific levels of effort and fatigue differed from one another.

## Results

Data-screening revealed that of the 6,750 decision-making trials, only 10 were invalid because participants did not respond (*n* = 5) or pressed an invalid key (*n* = 5). These trials were excluded from further analyses.

### Manipulation Checks

To test whether the fatigue manipulations evoked (domain-specific) subjective fatigue, two manipulation checks were performed. For an overview of all self-reported states before and after each experimental condition (see Table 1).

**TABLE 1:** Means and standard deviations of self-reported fatigue per condition and per measurement.

	**Cognitive fatigue (0–100)**		**Physical fatigue (0–100)**	
**Condition**	**Pre**	**Post**	**Pre**	**Post**
Cognitive fatigue	44.36 (23.07)	74.15 (22.39)	37.90 (23.25)	53.16 (26.74)
Physical fatigue	40.39 (29.85)	57.23 (29.96)	41.23 (26.71)	64.42 (30.01)
Control	37.66 (26.74)	34.96 (23.93)	38.96 (27.49)	37.28 (26.81)

*N = 20. Standard deviations are presented in parentheses.*

#### Subjective Cognitive Fatigue

In the first manipulation check, we compared the increases in self-reported cognitive fatigue between the cognitive fatigue, physical fatigue, and control condition. The main effect of condition was significant [χ^2^(2) = 21.763, *p* = 0.001] as well as the main effect of time [χ^2^(1) = 13.817, *p* = 0.001]. Crucially, also the interaction term Condition x Time was significant [χ^2^(2) = 11.612, *p* = 0.003], indicating that the increase in self-reported cognitive fatigue differed between the three conditions. Our confirmatory *post hoc* analysis compared the increases between the specific experimental conditions. This analysis revealed that subjective cognitive fatigue increased significantly more in the cognitive fatigue condition than in the control condition [*b* = 8.12, *SE* = 2.02, *t*(39.99) = 4.03, *p* < 0.001]. Interestingly, exploratory *post hoc* analyses revealed that the increase of cognitive fatigue was not significantly stronger in the cognitive fatigue condition than in the physical fatigue condition (*p* = 0.141) and that the increase in cognitive fatigue was significantly stronger in the physical fatigue condition in comparison to the control condition [*b* = 4.86, *SE* = 2.34, *t*(40) = 2.08, *p* = 0.045]. Another exploratory *post hoc* analysis revealed that subjective cognitive fatigue increased significantly in the cognitive fatigue condition [*b* = −29.78, *SE* = 6.76, *t*(95) = −4.405, *p* < 0.001] but not in the physical fatigue condition (*p* = 0.137) or in the control condition (*p* = 0.999). Finally, we explored the between-condition differences in self-reported fatigue before and after the experimental manipulations. This exploratory analysis revealed that before the manipulations, subjective cognitive fatigue was not significantly different between the three conditions (*p*’s > 0.05). After the manipulations, subjective cognitive fatigue was significantly higher in the cognitive fatigue condition in comparison to the control condition (*b* = −39.19, *SE* = 6.76, *t* = −5.796, *p* < 0.001) but not in comparison to the physical fatigue condition (*p* = 0.134). Moreover, subjective cognitive fatigue was also significantly higher in the physical fatigue condition compared to the control condition (*b* = −22.27, *SE* = 6.76, *t* = −3.294, *p* = 0.017). These analyses provide partial support for the success of the cognitive fatigue manipulation. Within the conditions, subjective cognitive fatigue increased in the cognitive fatigue condition, and not in the other conditions. However, the increase and level of subjective cognitive fatigue did not significantly differ between the cognitive fatigue and physical fatigue condition.

#### Subjective Physical Fatigue

In the second manipulation check, we tested whether self-reported physical fatigue increased more in the physical fatigue condition than in the cognitive fatigue and control condition. The main effect of condition was significant [χ^2^(2) = 10.347, *p* = 0.012] as well as the main effect of time [χ^2^(1) = 10.764, *p* = 0.002]. Crucially, also the interaction term Condition x Time was significant [χ^2^(2) = 7.818, *p* = 0.024], meaning that the increase in subjective physical fatigue differed between the three conditions. The confirmatory *post hoc* analysis revealed that subjective physical fatigue increased significantly more in the physical fatigue condition than in the control condition [*b* = 6.22, *SE* = 2.17, *t*(60) = 2.86, *p* = 0.006]. Surprisingly, exploratory analyses showed that the increase in subjective physical fatigue was not significantly stronger in the physical fatigue condition than in the cognitive fatigue condition (*p* = 0.337) and increased significantly more in the cognitive fatigue condition in comparison to the control condition [*b* = 4.24, *SE* = 1.96, *t*(40) = 2.16, *p* = 0.037]. Another exploratory analysis revealed that subjective physical fatigue increased in the physical fatigue condition [*b* = −23.19, *SE* = 6.46, *t*(95) = −3.56, *p* = 0.007] but not in the other two conditions (*p*’s > 0.05). Finally, we explored the between-condition differences in subjective physical fatigue before and after the experimental manipulations. These exploratory analyses revealed that before the experimental manipulations, subjective physical fatigue did not significantly differ between the three conditions (*p*’s > 0.05). However, after the manipulations, subjective physical fatigue was significantly higher in the physical fatigue condition compared to the control condition [*b* = −27.13, *SE* = 6.46, *t*(95) = −4.20, *p* < 0.001] but not in comparison to the cognitive fatigue condition (*p* = 0.508). Subjective physical fatigue was also not significantly higher in the cognitive fatigue condition than in the control condition (*p* = 0.148). These results again partially support the success of the manipulation. Subjective physical fatigue increased in the physical fatigue condition, but not in the other conditions. However, the increase and level of subjective physical fatigue did not significantly differ between the physical fatigue or cognitive fatigue condition.

From these manipulation checks, it follows that the fatigue manipulations were partially effective at inducing subjective fatigue. Significant increases in subjective fatigue were observed within the relevant conditions but the increases and post-measures did not significantly differ between the cognitive fatigue and physical fatigue conditions. Assuming that the single-item VAS-scales are valid, it is thus debatable whether we can test the impact of domain-specific fatigue on the decision to exert physical effort. To further evaluate the validity of the experimental tasks, we additionally looked into the (domain-specific) effort, frustration, boredom and stress participants reported (before and) after the experimental manipulations. Descriptive data of these experiences can be found in Supplementary Tables 1, 2. While we cannot be completely certain that the fatigue manipulations evoked the appropriate fatigue experiences, these data suggest that our manipulations did evoke the appropriate domain-specific demand experiences. As such, our findings will at the very least inform us about the impact of exerting (physical or cognitive) effort on subsequent physical effort-based decision-making.

### Main Analyses

In the main analysis, we tested whether the probability to accept physically effortful offers in the decision-making task was influenced by the physical effort requirements of offers, by the fatigue conditions and the interaction term Condition × Physical Effort Requirement. As expected, and confirming hypothesis 1, the analysis showed a significant effect of the physical effort requirements [χ^2^(1) = 37.536, *p* = 0.001]. *Post hoc* comparisons revealed that with each increase in physical effort requirement, the probability to accept offers was significantly lower (all *p*’s < 0.001). This replicates the longstanding law of least effort (Hull, 1943) which states that individuals tend to avoid effort when possible. Unexpectedly, no significant effect of condition was found (*p* = 0.253). Participants were not significantly less likely to accept offers after the cognitively or physically demanding task. Hypothesis 2 was thus rejected.

**FIGURE 2:**
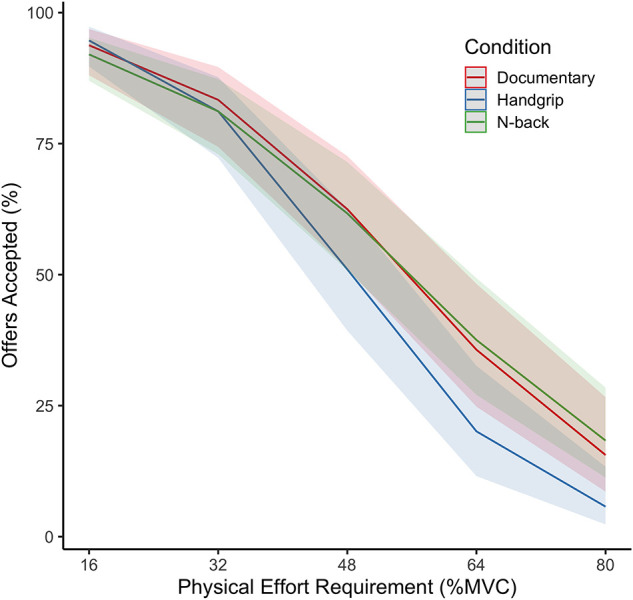
Effects of physical effort requirements and condition on percentage of offers accepted. *N* = 20. MVC, Maximum Voluntary Contraction. Shaded areas represent 95% confidence intervals. The overall percentage of effortful offers accepted did not differ significantly between the experimental conditions. However, the effort slope was significantly steeper after performing the handgrip exercise than after documentary watching or after performing the N-back task. Between-condition comparisons at the specific levels of physical effort requirement did not reach significance.

Most important, the interaction term Condition x Physical Effort Requirement significantly predicted the probability to accept physically effortful offers [χ^2^(2) = 0.017, *p* = 0.028]. In line with the prediction (hypothesis 3b), *post hoc* analyses as well as visual inspection of the interaction-effect (see Figure 2) revealed that the effort slope was significantly steeper in the physical fatigue condition than in the control condition (OR = 0.369, 95% CI [0.166, 0.737], *p* = 0.012). However, and against hypothesis 3a, the effort slope did not significantly differ between the cognitive fatigue condition and the control condition (*p* = 0.328). In fact, an exploratory analysis revealed that the effort slope was significantly steeper in the physical fatigue condition than in the cognitive fatigue condition (OR = 0.266, 95% CI [0.093, 0.663], *p* = 0.008). Between-condition comparisons at the specific levels of physical effort requirement did not reach significance (*p*’s > 0.05).

See Supplementary Material for additional data and analyses on reward sensitivity and performance on the execution task.

## Discussion

We aimed to investigate the impact of cognitive and physical fatigue on the decision-making process for exerting physical effort. We expected that cognitive and physical fatigue would increase the weight of effort costs within the cost-benefit analysis for the decision to exert physical effort. Despite thorough attempts to specifically manipulate cognitive and physical fatigue, the evidence for effective domain-specific fatigue manipulations was weak. While participants reported stronger increases in cognitive and physical fatigue in the fatiguing conditions than in the control condition, these increases did not differ between the two fatiguing conditions. In the remainder of this discussion, we will therefore be conservative and make no claims about the consequences of any specific forms of fatigue but rather focus on the impact of cognitive or physical effort exertion on subsequent effort-based decision-making.

The present experiment shows that people are sensitive to effort requirements, which validates the effort-based decision task. More important, exerting cognitive effort did not reduce the likelihood to accept physically effortful offers or strengthen the negative effort slope. This pattern of findings does not support Müller and Apps’s (2019) suggestion that prior effort exertion has domain-general effects on subsequent effort-based decision-making. Specifically, Müller and Apps (2019) argue that individuals would assign more weight to effort-costs after exerting effort, irrespective of the effort domain (i.e., physical or cognitive). If that were the case, performing a cognitively demanding task would increase the physical effort slope. The current study was the first to directly test this assumption and our findings do not support such a domain-general impact. Possibly, the cognitively demanding task was not sufficiently demanding to evoke domain-general effects on effort-based decision-making. Multiple studies have shown that the fatiguing effects of exerting cognitive effort depend on task difficulty rather than on time-on-task (Boksem and Tops, 2008; Chatain et al., 2019). While the 2-back task draws on multiple cognitive capacities such as working memory processing, updating and vigilance, the fixed task characteristics might not have been sufficiently demanding for our university sample to elucidate an impact on effort-based decision-making. Higher cognitive demands might be needed to evoke domain-general effects of cognitive effort exertion. A promising approach would be to adapt task difficulty to participant performance on a trial-by-trial basis, which has been shown to effectively evoke the phenomenology of cognitive effort (Lin et al., 2020). Establishing strong cognitive effort manipulations will be crucial to understand the impact of cognitive effort exertion on subsequent (physical) effort-based decision-making.

Interestingly, our results show that while physical effort exertion does not reduce the overall likelihood to accept physically effortful offers, it strengthens the effort slope. In line with findings of Iodice et al. (2017), participants weighted physical effort costs more heavily in the effort-based decision-making task after a demanding physical task. Importantly, we show that this effect not only applies to decisions about cycling duration but also about delivering physical force (i.e., squeezing). An important asset of the present study is that variation in effort levels of the high-effort options was not contaminated by the duration of physical effort requirements. While Iodice et al. (2017) manipulated effort levels by varying the duration of the high-effort options (i.e., 10–40 min of cycling), in the present study, exclusively the physical force requirements of high-effort options varied (i.e., 16–80% of participants’ MVC). As such, our study provides new evidence for the impact of physical effort exertion on the weighting of future physical effort-costs. It shows that physical effort-based decision-making is not fixed but depends on earlier bouts of physical effort exertion.

These findings provide interesting methodological and theoretical insights. Importantly, this was the first study to test the consequences of both cognitive and physical effort exertion for subsequent effort-based decision-making within a single study. Our findings show that physical effort decisions are sensitive to earlier bouts of effort exertion (i.e., a steeper effort slope after exerting physical effort). Crucially, the effect of physical effort exertion was larger than that of cognitive effort exertion, which did not significantly differ from the control condition. Our ability to show these differential consequences of effort exertion within the same experimental design is informative, even though these findings may be attributed to different reasons: It is possible that the predictions made in the neurocognitive framework of motivational fatigue (Müller and Apps, 2019) are incorrect and that cognitive fatigue does not affect the weight assigned to future effort-costs. If that is the case, an alternative explanation for lower levels of physical activity participation after cognitive exertion might be that the perception of effort increases as suggested by psychobiological models of endurance performance (e.g., Marcora, 2010; Pageaux, 2014; Martin et al., 2018), rather than the weight of effort-costs. Alternatively, the absence of evidence for a change in effort-costs could be ascribed to the methodological constraints of this study such as the relatively small sample size, the absence of clear domain-specific fatigue experiences and the very restricted form of physical effort exertion (i.e., hand squeezing), or the intensity of the cognitive task. Therefore, more research with larger samples, alternative effortful tasks and thorough manipulation checks will be needed to determine which mechanism explains the deterioration of physical performance after cognitive effort (Brown et al., 2020). The current study provides a strong methodological basis which can be drawn upon by future research to further investigate these processes.

At the same time, our findings are the first to provide direct support for Müller and Apps’s (2019) proposition that exerting physical effort increases the weight assigned to future effort-costs. That is, the negative impact of effort requirements on the likelihood to accept effortful offers (i.e., the effort slope) increased after earlier physical effort exertion. This finding provides a nuanced image of the consequences of physical effort exertion for the decision-making process with regard to subsequent effort. Individuals are not unwilling to exert any effort after earlier bouts of effort exertion (Thorndike, 1914) but they weight the effort-costs more strongly in the cost-benefit analyses underlying the decision to exert further effort or not. This could very well explain why, after performing an effortful task, individuals tend to perform worse on a subsequent effortful task, unless they are motivated to perform well by additional rewards (Boksem et al., 2006; Hopstaken et al., 2015, 2016). From the current perspective, this makes perfect sense. If the perceived costs of effort increase after a period of effort exertion while at the same time, additional rewards outweigh these increased costs, individuals will still engage in a subsequent effortful task. Here too, it will be valuable to investigate whether the observed changes in effort-based decision-making occur due to a change in the weight assigned to effort-costs (Müller and Apps, 2019), or due to a change in the perception of effort (Marcora, 2010; Pageaux, 2014; Martin et al., 2018).

With regard to the manipulation checks, it is important to note that the findings do not seem to align with the definition of cognitive fatigue as a state resulting from prolonged engagement in a cognitively demanding task (Boksem and Tops, 2008; Kanfer, 2011; van der Linden, 2011). If that were true, then the cognitively demanding task would have evoked more subjective cognitive fatigue on the single-item VAS-scale than the physically demanding task. The small sample size might account for the fact that the differences between the fatiguing conditions (see Table 1) failed to reach statistical significance (Button et al., 2013). Another reason for the absence of domain-specific fatigue differences might be that the specific type and level of cognitive and physical demands that the experimental tasks required (i.e., working-memory and local muscle performance) did not evoke the intended fatigue experiences. Crucially, the current manipulation issues also tap into the ongoing challenge to scientifically define fatigue and its (experimental) antecedents (van der Linden, 2011; Hockey, 2013). The multifaceted nature of fatigue (i.e., behavior, emotion, motivation, and information processing) makes it very hard to pinpoint the exact nature of fatigue (van der Linden, 2011). As outlined by Müller and Apps (2019), the same phenomenological experience of fatigue can occur after exerting effort into very different domains, which can make it very hard for people to differentiate between physical and cognitive fatigue on a single-item VAS-scale. Against this background, it is less surprising that our domain-specific fatigue manipulations did not result in convincing domain-specific differences in self-reported fatigue.

An important strength of the current study is its innovative and theory-driven design, which enabled us to test some of the core assumptions of dominant fatigue theories. Specifically, this was the first study in which the decision-making process for physical effort was assessed after bouts of both cognitive and physical effort exertion, which enabled us to compare the consequences of these specific forms of effort exertion. An interesting venue for future research will be to measure cognitive effort-based decision-making in addition to physical effort-based decision-making. While the present study provides a first glimpse into the domain-specificity of effort exertion on subsequent decision-making, adding cognitive effort-based decision-making will allow researchers to test the full range of domain specific and -general effects (i.e., all possible combinations within and between the physical and cognitive domain). A noteworthy example here is a study performed by Chong et al. (2018), in which the researchers used a single task to measure both cognitive- and physical effort-based decision-making. Similar tasks could very well be applied to further disentangle the effects of effort exertion, which can improve our understanding of both the antecedents and consequences of effortful behavior.

Despite its strong design, several important limitations should be stressed. As outlined before, an important limitation of this study is the absence of clear domain-specific fatigue manipulations. Subjective cognitive fatigue did not increase significantly more in the cognitive fatigue condition than in the physical fatigue condition and vice versa. This makes it impossible to draw definite conclusions about the impact of cognitive and physical fatigue on subsequent effort-based decision-making. Regarding the manipulation of cognitive fatigue, it will be valuable to select prolonged tasks that are more cognitively demanding (Pageaux and Lepers, 2018). While the n-back task has been used to induce cognitive fatigue before (Hopstaken et al., 2015, 2016), it might be more effective to use tasks that require other cognitive processes, such as inhibition (Smith et al., 2019). Moreover, researchers could select tasks that adapt to participants’ dispositional and situational cognitive capacities (for examples, see Lin et al., 2020; O’Keeffe et al., 2020), to ascertain that the task is similarly demanding for each participant. In a similar vein, it will be valuable to apply alternative tasks to induce physical fatigue in future research. We currently applied a task specifically requiring forearm muscle contraction and it might be valuable to use tasks requiring larger muscle mass (e.g., quadricep muscle contraction) or whole-body exercises (e.g., running or cycling) to induce physical fatigue. Another limitation was that we did not assess the perception of physical effort. Accumulating evidence suggests that the perception of effort plays a crucial role in explaining physical performance after cognitive effort exertion (Pageaux and Lepers, 2016, 2018; Brown et al., 2020) as well as in motor control and decision making in general (Cos, 2017; Wang et al., 2021). While participants in the present study did experience the different physical effort levels immediately after each manipulation task (i.e., within the familiarization task), including an assessment of perceived effort will be crucial for future research to obtain insight into its explanatory role for effort choices. Finally, the relatively small and homogeneous sample limits the generalizability of our conclusions. Twenty university students participated in our study and it would be interesting to see whether the current findings apply beyond this specific sample of university students. For example, it is possible that different effects would be observed in older individuals since both cognitive and physical capacities tend to deteriorate with age, which might affect the cost-benefit trade-offs people make. Moreover, inclusion of larger samples is recommended in future research. The current sample size resembled that of Iodice et al. (2017), who conducted a very similar study. However, larger samples will lead to more reliable estimates of effect sizes and increase the chances to obtain reproducible findings (Button et al., 2013).

To conclude, the present study advances our insight into the psychological mechanisms that underlie engagement in effortful behavior. While it was not possible to investigate the unique impact of cognitive and physical fatigue on subsequent physical effort-based decision-making, our study reveals very detailed consequences of effort exertion for subsequent effort decisions. Our findings confirm that individuals perceive physical effort to be costly and our results imply that individuals ascribe more weight to these physical effort-costs after prolonged exertion of physical but not cognitive effort. Individuals are thus not simply less likely to accept physically effortful offers after earlier bouts of effort exertion but this effect seems to depend upon the type of previous effort exertion as well as the specific effort levels of offers. Taking this specificity into account will help researchers to further improve our understanding of the psychology of physical activity, which could eventually contribute to the effectiveness of global physical activity promotion.

## Data Availability Statement

The datasets presented in this study can be found in online repositories. The names of the repository/repositories and accession number(s) can be found below: https://osf.io/zp7te/. This article has received the badges for Open Data, Open Materials, and Preregistration. More information about the Open Practices badges can be found at http://www.psychologicalscience.org/publications/badges.

## Ethics Statement

The studies involving human participants were reviewed and approved by the Ethics Committee Social Sciences of Radboud University. The patients/participants provided their written informed consent to participate in this study.

## Author Contributions

SA, DB, HV, MH, and SG designed the research. SA performed the research and analyzed the data. SA, DB, HV, MK, MH, and SG wrote the manuscript. All authors contributed to the article and approved the submitted version.

## Conflict of Interest

The authors declare that the research was conducted in the absence of any commercial or financial relationships that could be construed as a potential conflict of interest.

## Publisher’s Note

All claims expressed in this article are solely those of the authors and do not necessarily represent those of their affiliated organizations, or those of the publisher, the editors and the reviewers. Any product that may be evaluated in this article, or claim that may be made by its manufacturer, is not guaranteed or endorsed by the publisher.

## Footnotes

1 As we were primarily interested in the willingness to exert effort as a function of effort requirements, we did not formulate expectations about rewards. We did vary the reward levels in order to create incentive compatible offers without evoking heuristic responses (e.g., only accepting low-effort offers). Exploratory reward-analyses can be found in Supplementary Material.

2 For three participants, the actual order of sessions deviated from the original counterbalanced scheme.

3 We preregistered to only compare increases in subjective cognitive fatigue between the cognitive fatigue condition and the control condition to conserve statistical power. Also, for physical fatigue, we preregistered to only compare increases in subjective physical fatigue between the physical fatigue condition and the control condition. All other *post hoc* and omnibus comparisons were performed for completeness but these analyses should thus be considered exploratory.

4 Only data of the decision-making task were analyzed as the potential responses (i.e., squeeze or wait) within the actual execution task depended on the choices made within the decision-making task.

5 We preregistered to analyze our data separately in order to conserve statistical power for the effects that we were specifically interested in (i.e., the contrast between the two fatigue conditions and the control condition). The present study was meant to be a pilot study in a convenience sample (i.e., determined by a predefined time limit). As we could not predict the exact number of participants that would take part in our study within this time frame, we were conservative in our tests and restricted ourselves to analyses that exclusively tapped into the contrasts of interest (i.e., experimental conditions against control condition). Including all conditions in one analysis would result in more complex interactions, with more effects to estimate and resulting in a reduced statistical power. The omnibus test was performed for clarity and yields the same results as our preregistered *post hoc* analyses.
