# Supplementary material for: The Impact of Cognitive and Physical Effort Exertion on Physical Effort Decisions: A Pilot Experiment
Source: Front Psychol. 2021 Nov 2;12:645037. doi: 10.3389/fpsyg.2021.645037 (PMC8593226; doi:10.3389/fpsyg.2021.645037)
Supplement: Supplementary file 1 [file Data_Sheet_1.docx]

***Supplementary Material***

**Exploratory Analyses**

***Reward Sensitivity*** To investigate the impact of the cognitive fatigue manipulation on reward sensitivity, we tested a model predicting probability to accept physically effortful offers from condition (cognitive fatigue, physical fatigue, control), reward and the interaction term Condition x Reward. The analysis included a per-participant random adjustment to the fixed intercept (‘random intercept’) as well as a per-participant random adjustment to the fixed slope (‘random slope’). Post-hoc analyses were performed to obtain insight into specific differences between conditions.

 ***2-back Performance*** To better understand how performance on the 2-back task evolved over the course of the task, we performed two exploratory analyses. As an indicator of participants’ accuracy, we calculated d-prime per block of 111 trials as described by signal detection theory (Wickens, 2001). We first calculated the hit-rate, which is the number of correctly identified target trials (i.e., 2-back) divided by the total number of target trials. Next, we calculated the false alarm-rate, which is the number of non-targets that were incorrectly identified as targets, divided by the total number of non-targets. Finally, we subtracted the standardized false alarm-rate from the standardized hit-rate to calculate the d-prime. We then tested a mixed model in which d-prime was predicted by test block (1-8). This model also included a per-participant random adjustment to the fixed intercept (i.e., random intercept). In the second mixed model, we investigated whether trial number affected reaction times. This model included the fixed effect of trial number as well as the per-participant random adjustment to the fixed intercept (i.e., random intercept).

**Results Exploratory Analyses**

***Reward Sensitivity***The mixed model predicting choices from condition, reward and the interaction Condition x Reward did not show a significant effect of condition (*p* = .802). However, we found a significant effect of reward (χ^2^(1) = 33.266, *p* = .001). Post-hoc analyses showed that with every increase in rewards, participants were more likely to accept offers (*p*’s < .001). Moreover, also the interaction term Fatigue x Reward was significant (χ^2^(2) = 7.653, *p* = .022). Post-hoc analyses as well as visual inspection of the interaction effect (see Figure S1) revealed that the reward-slope was significantly less steep in the physical fatigue condition than in the control condition (OR = 0.962, 95% CI [0.933, 0.988], *p* = .006). However, the reward-slope did not significantly differ between the cognitive fatigue and control condition (*p* = .507) or between the cognitive fatigue and physical fatigue condition (*p* = .071). Finally, no between-condition differences were observed at the specific effort levels (*p*’s > .05). Thus, rewards indeed increased the probability to accept effortful offers. However, reward sensitivity only differed between the physical fatigue condition and the control condition.


***2-back Performance*** The mixed model testing the impact of test block on d-prime showed no significant effect of test block (*p* = .214). Thus, participant accuracy did not become significantly better or worse over the course of the 2-back task. The average d-prime did indeed not change much from block 1 (*M* = 2.12, *SD* = 0.69) to block 8 (*M* = 2.17, *SD* = 1.22). Interestingly, our second analysis regarding 2-back performance showed that trial number significantly predicted reaction times (*b* = -.0002, *SE* = 0.000009, *t*(17910.08) = -17.18, *p* < .001). Thus, participants became faster at responding throughout the 2-back task.

**Figure S1.** *The (interaction) effects of reward and condition on accepted offers.*
*Note*. N = 20. Shaded areas represent 95% confidence intervals. The reward slope was always significantly positive but was significantly less steep after performing the handgrip exercise compared to the other two conditions. No main effect of condition was found.


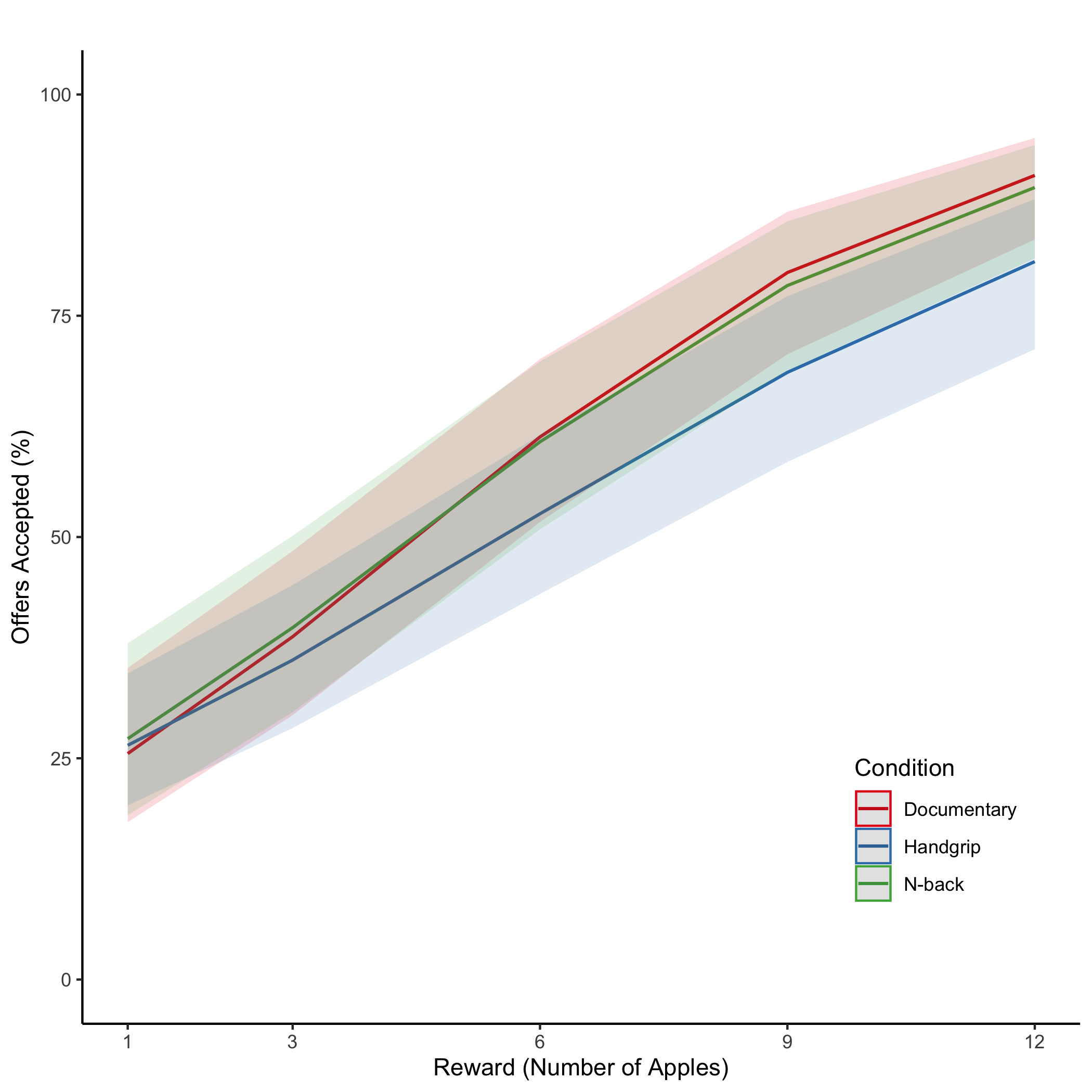


**Table S1**

|  |  | General effort^a^ | |  | Mental demand^a^ | |  | Physical demand^a^ | |  | Frustration^a^ | |
| --- | --- | --- | --- | --- | --- | --- | --- | --- | --- | --- | --- | --- |
| Condition |  | Mean | SD |  | Mean | SD |  | Mean | SD |  | Mean | SD |
| Cognitive fatigue |  | 61.92 | 30.08 |  | 82.12 | 19.95 |  | 25.24 | 29.36 |  | 67.81 | 27.69 |
| Physical fatigue |  | 72.86 | 26.38 |  | 29.79 | 26.51 |  | 85.50 | 14.24 |  | 67.32 | 17.12 |
| Control |  | 20.05 | 26.50 |  | 23.20 | 24.05 |  | 8.36 | 13.69 |  | 6.09 | 11.54 |

*Means and standard deviations of experienced (domain-specific) demands and frustration per condition.*

*Note.* N = 20.
^a^General effort, mental demand, physical demand and frustration were all measured with single-item VAS-scales (ranging from “Not at all” to “Extremely”: ‘How effortful/mentally demanding/physically demanding/frustrating was the task?’).

**Table S2**

|  |  | Stress^a^ | |  | Boredom^a^ | |
| --- | --- | --- | --- | --- | --- | --- |
| Condition |  | Pre | Post |  | Pre | Post |
| Cognitive fatigue |  | 43.54 (23.78) | 41.36 (22.26) |  | 44.63 (22.63) | 81.69 (25.61) |
| Physical fatigue |  | 36.34 (23.40) | 38.38 (23.14) |  | 42.23 (22.43) | 79.69 (20.76) |
| Control |  | 41.72 (26.70) | 15.92 (17.46) |  | 32.63 (17.94) | 32.27 (24.50) |

*Means and standard deviations of experienced stress and boredom before and after the manipulations.*

*Note.* N = 20. Standard deviations are presented in parentheses.
^a^Stress and boredom were both measured with single-item VAS-scales (ranging from “Not at all” to “Extremely”: ‘How stressed/bored do you currently feel?’).

**Table S3**

|  |  | % Accept-trials | |  | % Successful performance | |
| --- | --- | --- | --- | --- | --- | --- |
| Effort level (%MVC) |  | Mean | SD |  | Mean | SD |
| 16 |  | 87.27 | 33.36 |  | 99.58 | 33.36 |
| 32 |  | 80.18 | 39.90 |  | 98.64 | 39.90 |
| 48 |  | 60.36 | 48.96 |  | 94.89 | 48.96 |
| 64 |  | 35.57 | 47.92 |  | 82.14 | 47.92 |
| 80 |  | 18.36 | 38.75 |  | 68.32 | 38.75 |

*Apple-gathering trials and performance per effort level.*

*Note.* N = 20. % Accept-trials = percentage of apple-gathering trials on which participants could squeeze to obtain a reward; % Successful performance = percentage of accept-trials on which participants actually squeezed to obtain a reward.

**References**

Wickens, T. D. (2001). *Elementary Signal Detection Theory*. Oxford University Press. http://dx.doi.org/10.1093/acprof:oso/9780195092509.001.0001
